# Histogram Analysis and Visual Heterogeneity of Diffusion-Weighted Imaging with Apparent Diffusion Coefficient Mapping in the Prediction of Molecular Subtypes of Invasive Breast Cancers

**DOI:** 10.1155/2019/2972189

**Published:** 2019-11-22

**Authors:** Joao V. Horvat, Aditi Iyer, Elizabeth A. Morris, Aditya Apte, Blanca Bernard-Davila, Danny F. Martinez, Doris Leithner, Olivia M. Sutton, R. Elena Ochoa-Albiztegui, Dilip Giri, Katja Pinker, Sunitha B. Thakur

**Affiliations:** ^1^Department of Radiology, Breast Imaging Service, Memorial Sloan Kettering Cancer Center, 300 E 66^th^ Street, New York, NY 10065, USA; ^2^Department of Medical Physics, Memorial Sloan Kettering Cancer Center, 1275 York Ave, New York, NY 10065, USA; ^3^Department of Diagnostic and Interventional Radiology, University Hospital Frankfurt, Theodor-Stern-Kai 7, 60590 Frankfurt, Germany; ^4^Department of Pathology, Memorial Sloan Kettering Cancer Center, 1275 York Ave, New York, NY 10065, USA; ^5^Department of Biomedical Imaging and Image-Guided Therapy, Molecular and Gender Imaging Service, Medical University of Vienna, Waehringer Guertel 18-20, 1090 Vienna, Austria

## Abstract

**Objective:**

To investigate if histogram analysis and visually assessed heterogeneity of diffusion-weighted imaging (DWI) with apparent diffusion coefficient (ADC) mapping can predict molecular subtypes of invasive breast cancers.

**Materials and Methods:**

In this retrospective study, 91 patients with invasive breast carcinoma who underwent preoperative magnetic resonance imaging (MRI) with DWI at our institution were included. Two radiologists delineated a 2-D region of interest (ROI) on ADC maps in consensus. Tumors were also independently classified into low and high heterogeneity based on visual assessment of DWI. First-order statistics extracted through histogram analysis within the ROI of the ADC maps (mean, 10th percentile, 50th percentile, 90th percentile, standard deviation, kurtosis, and skewness) and visually assessed heterogeneity were evaluated for associations with tumor receptor status (ER, PR, and HER2 status) as well as molecular subtype.

**Results:**

HER2-positive lesions demonstrated significantly higher mean (*p*=0.034), Perc50 (*p*=0.046), and Perc90 (*p*=0.040), with AUCs of 0.605, 0.592, and 0.652, respectively, than HER2-negative lesions. No significant differences were found in the histogram values for ER and PR statuses. Neither quantitative histogram analysis based on ADC maps nor qualitative visual heterogeneity assessment of DWI images was able to significantly differentiate between molecular subtypes, i.e., luminal A versus all other subtypes (luminal B, HER2-enriched, and triple negative) combined, luminal A and B combined versus HER2-enriched and triple negative combined, and triple negative versus all other types combined.

**Conclusion:**

Histogram analysis and visual heterogeneity assessment cannot be used to differentiate molecular subtypes of invasive breast cancer.

## 1. Introduction

Breast cancer classification according to tumor molecular subtype is nowadays routinely performed and is used to predict cancer aggressiveness and to guide recommendations for systemic treatments. Breast cancer can be classified into four molecular subtypes (luminal A, luminal B, human epidermal growth factor receptor 2- (HER2-) enriched, and triple negative) that present with distinctly different prognoses and treatment responses [1, 2]. Less aggressive molecular subtypes such as luminal A cancers (which are the most common type of breast cancer) are often low grade, susceptible to antihormonal therapy and have a better prognosis [3]. More aggressive molecular subtypes such as triple negative and HER2-enriched cancers have a propensity for metastatic disease and thus require treatment with either cytotoxic chemotherapy or the combination of cytotoxic chemotherapy and targeted anti-HER2 treatment [4–6]. In addition to molecular subtypes, intratumoral heterogeneity, i.e., the presence of cell clones of different levels of aggressiveness within one lesion, has been linked to tumor aggressiveness and poor prognosis [7].

To date, breast cancer classification according to molecular subtypes and initial treatment decisions are made based on breast biopsy. However, biopsy provides only a snapshot of the tumor biology and is subject to selection bias. In addition, as more and more tumors are being treated with either neoadjuvant cytotoxic or endocrine treatment, it is increasingly important to have the ability to achieve an accurate assessment of tumor biology in the preoperative setting [8].

Advances in imaging technology have allowed for the use of multiparametric features of magnetic resonance imaging (MRI) to improve breast cancer detection and characterization [9–11]. Amongst the investigated functional parameters, diffusion-weighted imaging (DWI) has emerged as one of the most important and easily obtainable multiparametric imaging features [12]. The quantification of DWI with apparent diffusion coefficient (ADC) mapping may demonstrate tumor characteristics that enable the noninvasive assessment of prognosis and tumor behavior preoperatively [13, 14].

Tumor molecular subtypes present with different vascularity and cellularity which are related to their underlying receptor status, especially in relation to estrogen receptor (ER) and HER2 status. The differences in vascularity and cellularity can affect the signal intensity of the tumor on DWI and may also affect tumor heterogeneity on DWI [15, 16]. Histogram analysis has been proposed as a quantitative method to evaluate the distribution of DWI and ADC values within a designated region of interest (ROI), with the potential of characterizing the amount of heterogeneity in a tumor [17, 18]. Patterns of value distribution on histogram analysis might be different amongst tumors with distinct biology [19]. In addition, tumor heterogeneity detected on histogram analysis may be visible on DWI, and yet to date, the visual assessment of heterogeneity on DWI has not been investigated for its usefulness to predict tumor characteristics.

In this context, the objective of our study was to evaluate if histogram analysis of DWI with ADC mapping can be used to predict molecular subtypes of invasive breast cancers and if visual assessment of tumor heterogeneity on DWI can outperform histogram analysis in the prediction of molecular subtypes.

## 2. Materials and Methods

### 2.1. Patients

In this health insurance portability and accountability act-compliant and institutional review board-approved retrospective study, we queried our institutional database for consecutive patients with invasive ductal or lobular carcinoma of the breast who underwent preoperative 3.0T MRI with dynamic contrast-enhanced (DCE) and DWI at our institution from January 2011 to January 2013. There were 188 patients who matched our search criteria. The exclusion criteria were as follows: (1) lesion smaller than 1 cm (*n* = 15); (2) previous treatment for breast cancer (*n* = 37); (3) unavailable receptor status on the pathology report (*n* = 10); and (4) poor image quality of DWI (*n* = 35). The final study population consisted of 91 patients. The need for informed consent was waived by the institutional review board.

### 2.2. MRI Studies

The MRI studies were performed using a 3.0T Discovery MR750 equipment (GE Healthcare, Milwaukee, WI, USA) with a dedicated 16-channel phased-array breast coil (Sentinelle Vanguard, Toronto, Canada). The standard multiparametric breast protocol was performed: axial T2-weighted imaging with and without fat saturation, DWI with ADC mapping, and DCE before and at 3 timepoints at 60 s intervals after administration of contrast media (gadopentetate dimeglumine given at 0.1 mmol/kg). The DWI sequence parameters were as follows: 2D single-shot, dual spin echo-planar imaging sequences (TR 6000 ms; minimum TE; flip angle 90°); acquisition matrix: 98 × 98 or 128 × 128; reconstructed matrix 256 × 256; FOV 28–38 cm; slice thickness: 4 or 5 mm; NEX 3; slice gap: 0-1 mm; fat suppression: enhanced; parallel imaging: ASSET; acquisition time approximately 2 min for 2 b-values: 0 and 1000 s/mm^2^.

All DWI data were transferred to a computer, and an in-house program prepared using MATLAB version 7.14 (MathWorks, Natick, MA) was used to generate ADC parametric maps.

### 2.3. Image Evaluation

Two breast radiologists (^xx^) with 8 and 13 years of experience in breast MRI reviewed the MRI studies. The largest invasive tumor in each patient was identified on DCE images and correlated with DWI, and subsequently the slice with the largest tumor diameter on high b-value images (1000 s/mm^2^) was selected. A two-dimensional ROI was drawn on the ADC map in consensus using the mouse cursor in a free hand fashion to mark the lesion boarders. The ROI included as much of the tumor as possible while the cystic areas, areas of normal breast parenchyma, and biopsy markers were avoided whenever possible. In a second step, the radiologists independently classified all tumors into two categories (low vs. high heterogeneity) based on the visual assessment of tumor heterogeneity on DWI high b-value (1000 s/mm^2^) images. The visual classification between low and high heterogeneity was done subjectively and based solely on the radiologists' experience and judgement.

### 2.4. Histopathology

Tumor histopathology was reviewed by a dedicated pathologist (xx) with 30 years of experience. The tumors were classified according to molecular subtype based on hormone receptor and HER2 status. The immunohistochemistry results as obtained from surgical specimens were considered the reference standard. In patients who underwent neoadjuvant chemotherapy after the MRI study, the results obtained from the biopsy specimen were used. Tumors were classified as luminal A if the specimen was estrogen receptor (ER) or progesterone receptor (PR) positive and HER2 negative; luminal B if the specimen was ER or PR positive and HER2 positive; HER2-enriched if the specimen was ER and PR negative and HER2 positive; and triple negative if the specimen was ER, PR, and HER2 were negative, as described in the previous studies [1, 2]. The HER2 status was considered negative if the staining was 0 or 1+, equivocal if it was 2+, and positive if it was 3+. Tumors with equivocal HER2 status were evaluated using fluorescence in situ hybridization and considered positive if HER2 gene amplification was observed and negative if no gene amplification was observed.

### 2.5. Statistical Analysis

All statistical analyses were performed with SAS version 9.4 (the SAS Institute Inc., Cary, NC, USA). Metric data values were expressed as mean or percentage values, as appropriate. Differences in first order statistics of histogram values between molecular subtypes were assessed for significance using the Wilcoxon rank sum and Mann–Whitney *U* tests, as appropriate. Comparison was performed between luminal A versus all other subtypes (luminal B, HER2-enriched, and triple negative combined), between luminal A and B combined versus HER2-enriched and triple negative combined, and between triple negative versus all other subtypes (luminal A, luminal B, and HER2-enriched combined). The first-order statistics assessed were as follows: the mean; 10th (Perc10), 50th (Perc50), and 90th (Perc90) percentiles; standard deviation; kurtosis; and skewness. *p* values <0.05 were considered statistically significant. The receiver operating characteristic curve was generated using MATLAB version 7.14 (MathWorks, Natick, MA). Associations between visual heterogeneity (low vs. high heterogeneity) and molecular subtype were also analyzed. The agreement between the two readers on visual assessment was quantified, and coefficient values closer to 1 were indicative of better agreement.

## 3. Results

### 3.1. Population

The mean patient age was 48 years (range, 27–68). The mean tumor size was 3.5 cm (range, 1–16.6 cm). There were 70 (76.9%) masses and 21 (23.1%) nonmass enhancements. There were 49 (53.8%) luminal A, 8 (8.8%) luminal B, 11 (12.1%) HER2-enriched, and 23 (25.3%) triple negative tumors.

### 3.2. Histogram Values and Receptor Status

**Figure 1:**
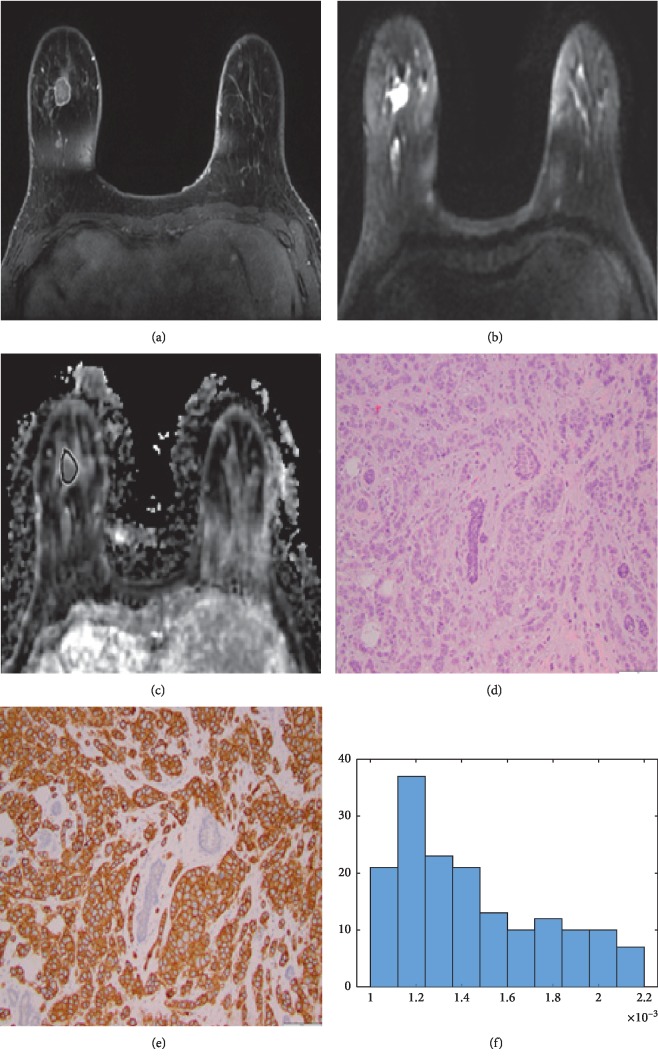
43-year-old woman with human epidermal growth factor receptor 2- (HER2-) positive invasive ductal carcinoma of the right breast on dynamic contrast-enhanced magnetic resonance imaging (DCE-MRI) (a), diffusion-weighted imaging (DWI) (b), apparent diffusion coefficient (ADC) map (c), histopathology (d), and HER2 staining (e). The histogram obtained (f) displayed a peak around 1.2 × 10^−3^ mm^2^/s and non-Gaussian distribution, in spite of being classified as low heterogeneity on visual assessment.

**Figure 2:**
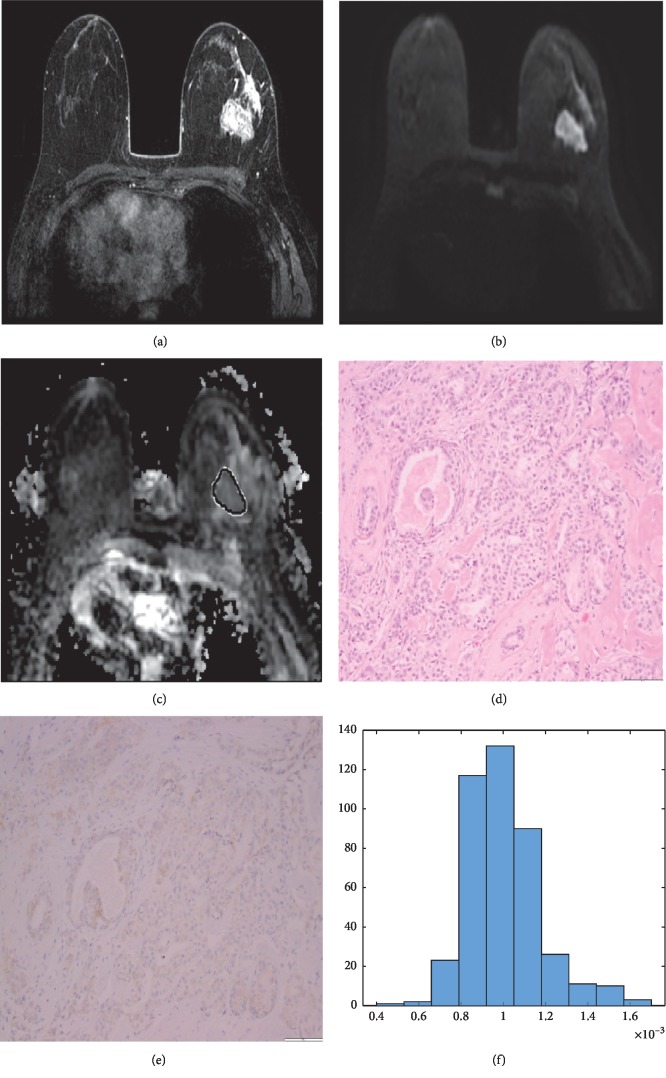
55-year-old woman with human epidermal growth factor receptor 2- (HER2-) negative invasive ductal carcinoma of the left breast on dynamic contrast-enhanced magnetic resonance imaging (DCE-MRI) (a), diffusion-weighted imaging (DWI) (b), apparent diffusion coefficient (ADC) map (c), histopathology (d), and HER2 staining (e). The histogram obtained (f) displayed a peak around 1.0 × 10^−3^ mm^2^/s and Gaussian distribution, in spite of being classified as high heterogeneity on visual assessment.

Significant differences were found in the histogram values between HER2 positive and HER2 negative tumors: mean (*p*=0.034), Perc50 (*p*=0.046), and Perc90 (*p*=0.040), with areas under the curve (AUCs) of 0.605, 0.592, and 0.652, respectively. HER2 positive tumors had higher ADC values than HER2 negative: mean 1.25 × 10^−3^ vs 1.12 × 10^−3^ mm^2^/s, Perc50 1.23 × 10^−3^ vs 1.10 × 10^−3^ mm^2^/s, and Perc90 1.62 × 10^−3^ vs 1.43 × 10^−3^ mm^2^/s, respectively. There were no significant differences in the histogram values between HER2 positive and HER2 negative tumors in terms of Perc10 (*p*=0.101), standard deviation (*p*=0.165), kurtosis (*p*=0.815), and skewness (*p*=0.944). Case examples of HER2 positive and HER2 negative tumors are demonstrated in Figures 1 and 2.

There were no significant differences in the histogram values between positive and negative ER or between positive and negative PR: mean (*p*=0.096 and 0.232), Perc10 (*p*=0.113 and 0.137), Perc50 (*p*=0.095 and 0.223), Perc90 (*p*=0.142 and 0.424), standard deviation (*p*=0.603 and 0.866), kurtosis (*p*=0.888 and 0.828), and skewness (*p*=0.505 and 0.871). The results of histogram analysis in regard to receptor status are demonstrated in Table 1.

### 3.3. Histogram Values and Molecular Subtypes

No significant differences were found in the histogram values between luminal A cancers and all the other types combined: mean (*p*=0.204), Perc10 (*p*=0.216), Perc50 (*p*=0.237), Perc90 (*p*=0.149), standard deviation (*p*=0.222), kurtosis (*p*=0.494), and skewness (*p*=0.896). No significant differences were found in the histogram values for luminal A and B combined versus HER2-enriched and triple negative combined: mean (*p*=0.204), Perc10 (*p*=0.130), Perc50 (*p*=0.115), Perc90 (*p*=0.167), standard deviation (*p*=0.081), kurtosis (*p*=0.941), and skewness (*p*=0.574). Similarly, no significant differences were found between triple negative tumors and all other subtypes combined: mean (*p*=0.604), Perc10 (*p*=0.915), Perc50 (*p*=0.636), Perc90 (*p*=0.485), standard deviation (*p*=0.479), kurtosis (*p*=0.574), and skewness (*p*=0.931). The results of the histogram analysis in regard to breast cancer molecular subtype are demonstrated in Table 2.

### 3.4. Visual Heterogeneity and Molecular Subtypes

No significant associations were found between visual heterogeneity and molecular subtype (Table 3). When comparing luminal A cancers versus all other types combined, *p* values of 0.300 for reader 1 and 0.538 for reader 2 were obtained. When comparing luminal A and B combined versus HER2-enriched and triple negative combined, *p* values of 0.603 for reader 1 and 0.682 for reader 2 were observed. Similarly, no significant difference was observed when comparing triple negative tumors with all other molecular subtypes combined, with *p* values of 0.133 for reader 1 and of 0.960 for reader 2. There was an almost perfect agreement between the two readers while classifying lesions into low or high heterogeneity (*κ* = 0.82).

## 4. Discussion

In this study, we investigated if histogram analysis and visually assessed heterogeneity of DWI with ADC mapping can be used to predict the molecular subtype of invasive breast cancers. First-order histogram analysis of ADC values showed that there were significant associations in histogram values of the mean, Perc50, and Perc90 values with HER2 status whereas no significant associations were found in histogram values with tumor ER and PR status. First-order histogram analysis was not able to accurately predict molecular subtype in the comparison of luminal A versus all other subtypes combined, luminal A and B combined versus HER2-enriched and triple negative combined, and triple negative versus all other subtypes combined. Likewise, visually assessed heterogeneity on DWI could not predict molecular subtypes of breast cancer.

The use of ADC as a tool for the differentiation between benign and malignant lesions has been widely explored in several studies. Malignant tumors usually have lower ADC values than benign lesions due to high cellularity [20, 21]. Likewise, invasive ductal carcinomas have lower ADC values than ductal carcinomas in situ [22]. ADC has been also investigated for the prediction of prognostic factors, such as positive axilla and lymphovascular invasion, with promising results [20, 21, 23–25].

The assessment of receptor status using different ADC metrics has led to conflicting results in the literature [16, 24, 26–29]. While some studies demonstrated lower ADC values for ER and PR positive tumors and higher for HER2 positive lesions, others did not find any significant associations between ADC measurements and receptor status. In our study, no significant differences were found in first-order histogram values regarding ER and PR status. Mean, Perc10, Perc50, and Perc90 values were lower for ER positive tumors, in line with the previous studies, but this difference was not statistically significant. On the other hand, mean, Perc50, and Perc90 values were significantly higher for HER2 positive tumors. This can be explained by the fact that HER2 positive tumors may present higher neovascularity than HER2 negative tumors; the higher ADC values in tumors with high neovascularity is caused by an increased plasma permeability that is observed in these blood vessels [16, 30–34]. Although our results demonstrate that HER2 status may be assessed using ADC histogram values, the AUCs reflected moderate accuracy. It has been noted in a previous study that the accuracy of predicting HER2 status using ADC is much lower than that of differentiating benign from malignant lesions [35].

Molecular subtype classification of breast cancer based on immunohistochemistry receptor status has significant importance in clinical practice [1, 36]. Receptor status guides the administration of hormone, HER2 targeted, and neoadjuvant therapies. Molecular subtypes are also used as prognostic factors for survival and recurrence. Although HER2 positive lesions may present higher ADC values, significant overlap occurs when separating lesions into the 4-category molecular subtype classification. In our study, the results obtained from histogram analysis of ADC were not able to differentiate molecular subtypes. A previous study demonstrated that maximum values of ADC could be used to differentiate molecular subtypes of breast cancer [26]. Although the software used in our study did not specifically evaluate maximum ADC values, the mean, Perc10, Perc50, and Perc90 results obtained were lower for luminal cancers in comparison with other subtypes, but this difference was not statistically significant.

Tumor heterogeneity is frequently associated with malignancy, aggressiveness, and response to treatment [19]. Different measurements obtained from histogram analysis may assess heterogeneity, such as kurtosis (flatness of a histogram) and skewness (asymmetry of a histogram) [17, 37]. Since intratumoral heterogeneity is often already present on visual assessment of DWI, we investigated if the classification of tumors into low and high heterogeneity on DWI can be used to predict molecular subtypes of breast cancer, with disappointing results. Similar to histogram analysis results, the visual assessment of tumor heterogeneity on DWI cannot predict the molecular subtype.

In a recent study, Suo et al. [38] demonstrated that entropy, which is also associated with heterogeneity, may be used to differentiate between luminal A tumors and other molecular subtypes on ADC map with moderate sensitivity and specificity. Although we did not use this feature in our data analysis, we believe that these promising results may be the basis of future studies that may reduce the significant overlap on the values obtained on ADC maps of tumors with different biological behavior.

Our study has a few limitations. First, lesions smaller than 1 cm were not included in the study. Second, only first-order statistics were considered and whole tumor segmentation was not performed as it is time-consuming and less likely to be used in clinical practice. Third, there was no standardization on the classification by visual assessment of tumor heterogeneity, which was based solely on the radiologist's subjective judgement. Lastly, molecular subtype classification was based on immunohistochemistry surrogates as genetic analysis was not performed.

## 5. Conclusions

In conclusion, neither histogram analysis of ADC values nor visually assessed heterogeneity on DWI can be used to predict molecular subtypes of breast cancer. Although the mean, Perc50, and Perc90 ADC values were significantly higher for HER2-positive than HER2-negative tumors, the accuracy for this differentiation is suboptimal and not ready for clinical use. Further large-scale studies are necessary to investigate if histogram analysis can play a role within radiomics analysis for the classification of invasive breast tumors.

## Figures and Tables

**Table 1:** Comparison of the average values of apparent diffusion coefficient (ADC) (mm^2^/s) on histogram analysis according to receptor status.

	ER+	ER−	*p*	PR+	PR−	*p*	HER2+	HER2−	*p*
Mean	1.11	1.20	0.096	1.12	1.18	0.232	1.25	1.12	**0.034**
Perc10	0.82	0.90	0.113	0.82	0.89	0.137	0.92	0.60	0.101
Perc50	1.09	1.19	0.095	1.10	1.17	0.223	1.23	1.10	**0.046**
Perc90	1.43	1.54	0.142	1.45	1.51	0.424	1.62	1.43	**0.040**
SD	0.24	0.25	0.603	0.24	0.24	0.866	0.27	0.23	0.165
Kurtosis	3.26	3.22	0.888	3.22	3.28	0.828	3.30	3.23	0.815
Skewness	0.33	0.24	0.505	0.31	0.29	0.871	0.29	0.30	0.944

ER: estrogen receptor; HER2: human epidermal growth factor receptor 2; Perc: percentile; PR: progesterone receptor; SD: standard deviation.

**Table 2:** Comparison of the average values of ADC (mm^2^/s) on histogram analysis according to molecular subtype.

	Luminal A	Others	*p*	Luminal A/B	Others	*p*	Triple negative	Others	*p*
Mean	1.12	1.18	0.204	1.12	1.19	0.204	1.12	1.15	0.604
Perc10	0.82	0.88	0.216	0.82	0.89	0.130	0.84	0.85	0.915
Perc50	1.10	1.16	0.237	1.10	1.17	0.115	1.11	1.14	0.636
Perc90	1.44	1.51	0.149	1.44	1.52	0.167	1.43	1.49	0.485
SD	0.24	0.25	0.222	0.24	0.24	0.081	0.23	0.25	0.479
Kurtosis	3.16	3.34	0.494	3.25	3.23	0.941	3.37	3.20	0.574
Skewness	0.31	0.29	0.896	0.33	0.24	0.574	0.29	0.30	0.931

Perc: percentile; SD: standard deviation.

**Table 3:** Visual classification of molecular subtypes of breast cancer into low and high heterogeneity on diffusion-weighted imaging (DWI) by the two readers.

Molecular subtypes	Reader 1		Reader 2	
	Low heterogeneity	High heterogeneity	Low heterogeneity	High heterogeneity
Luminal A	24 (49.0%)	25 (51.0%)	23 (46.9%)	26 (53.1%)
Luminal B	4 (50%)	4 (50%)	3 (37.5%)	5 (62.5%)
HER2-enriched	5 (45.5%)	6 (54.5%)	4 (36.4%)	7 (63.6%)
Triple negative	7 (30.4%)	16 (69.6%)	10 (43.5%)	13 (56.5%)

HER2: human epidermal growth factor receptor 2.

## Data Availability

The patient data used to support the findings of this study have not been made available because of patient privacy protection.
